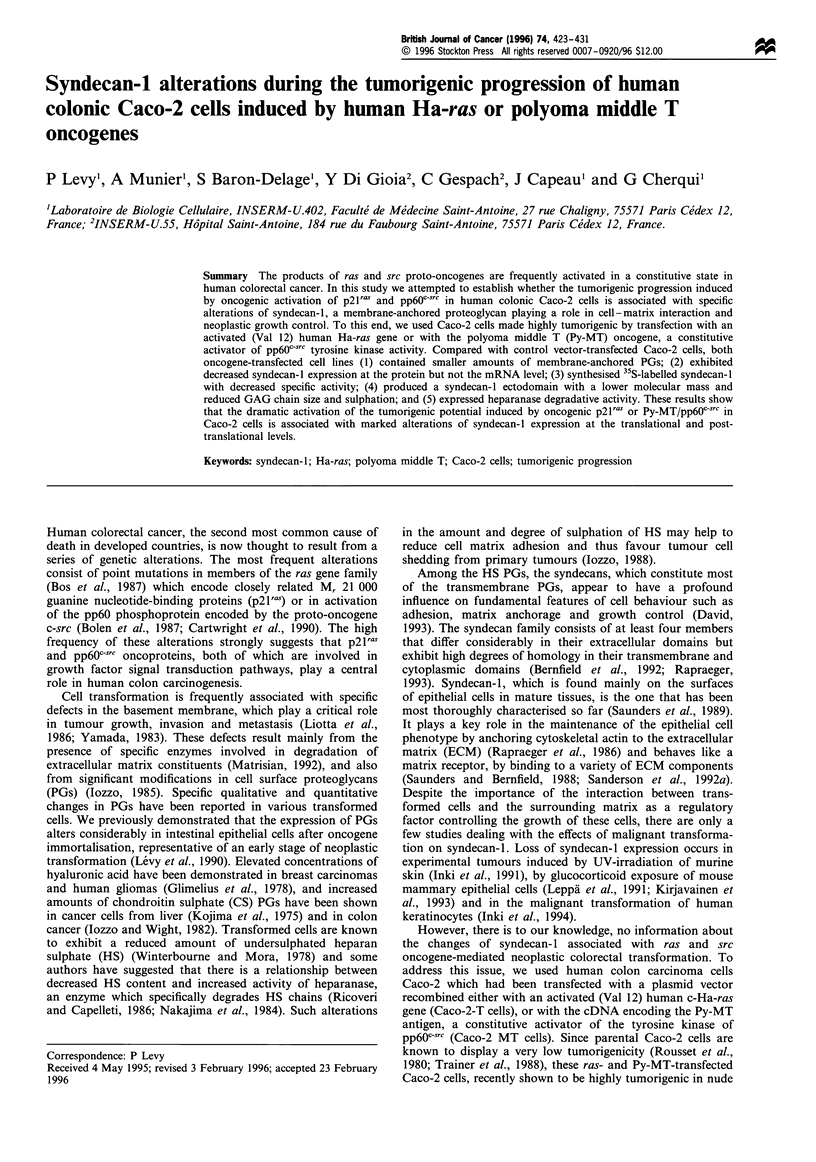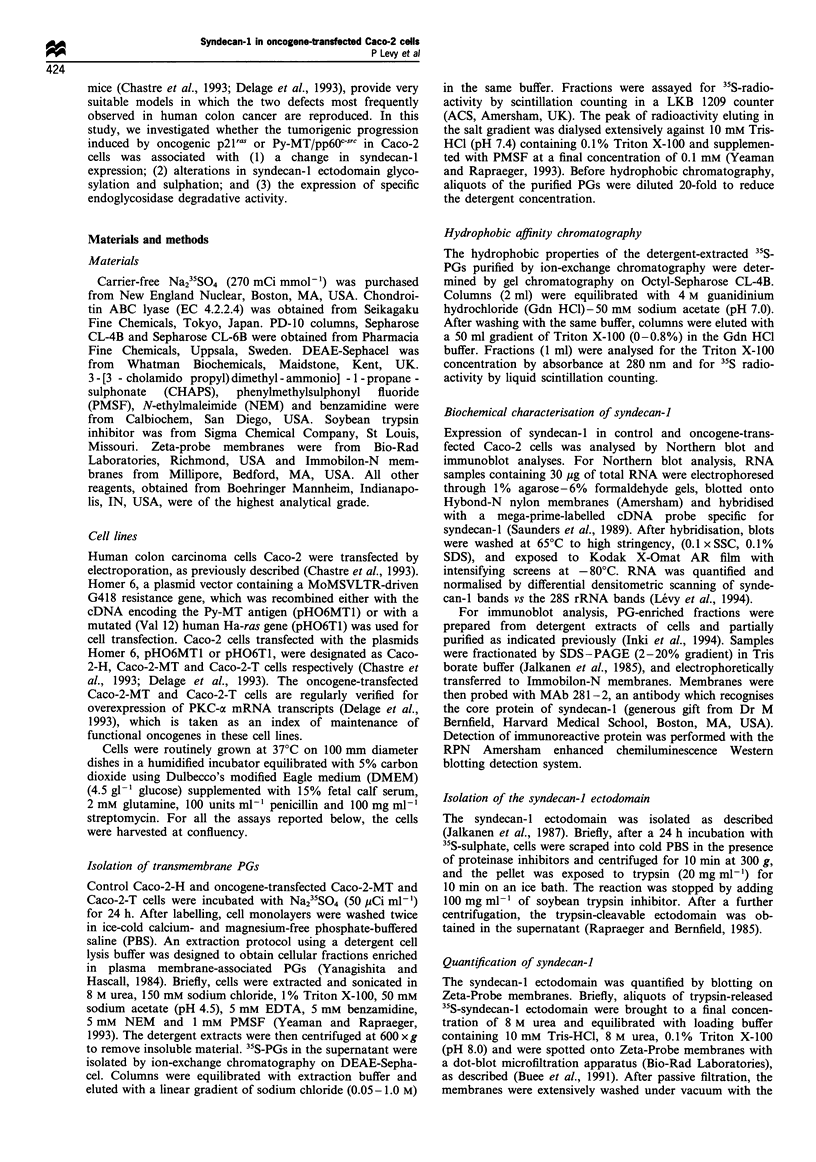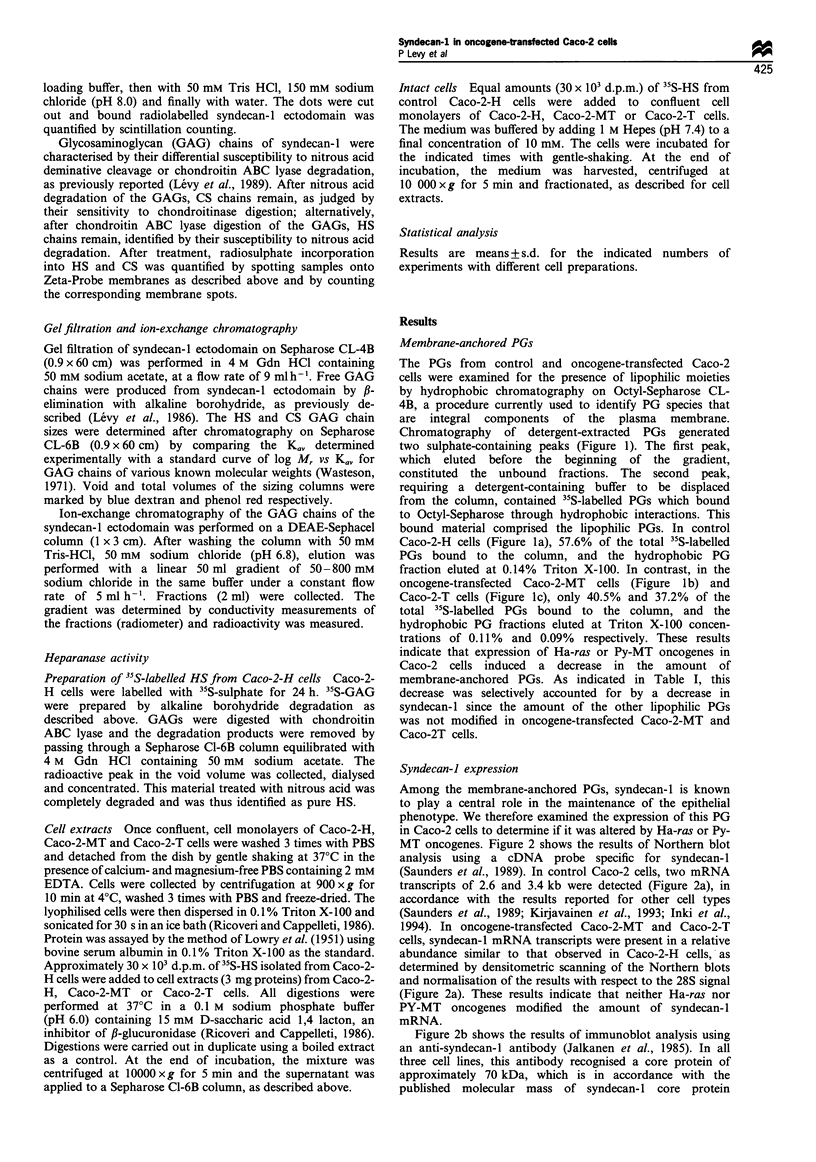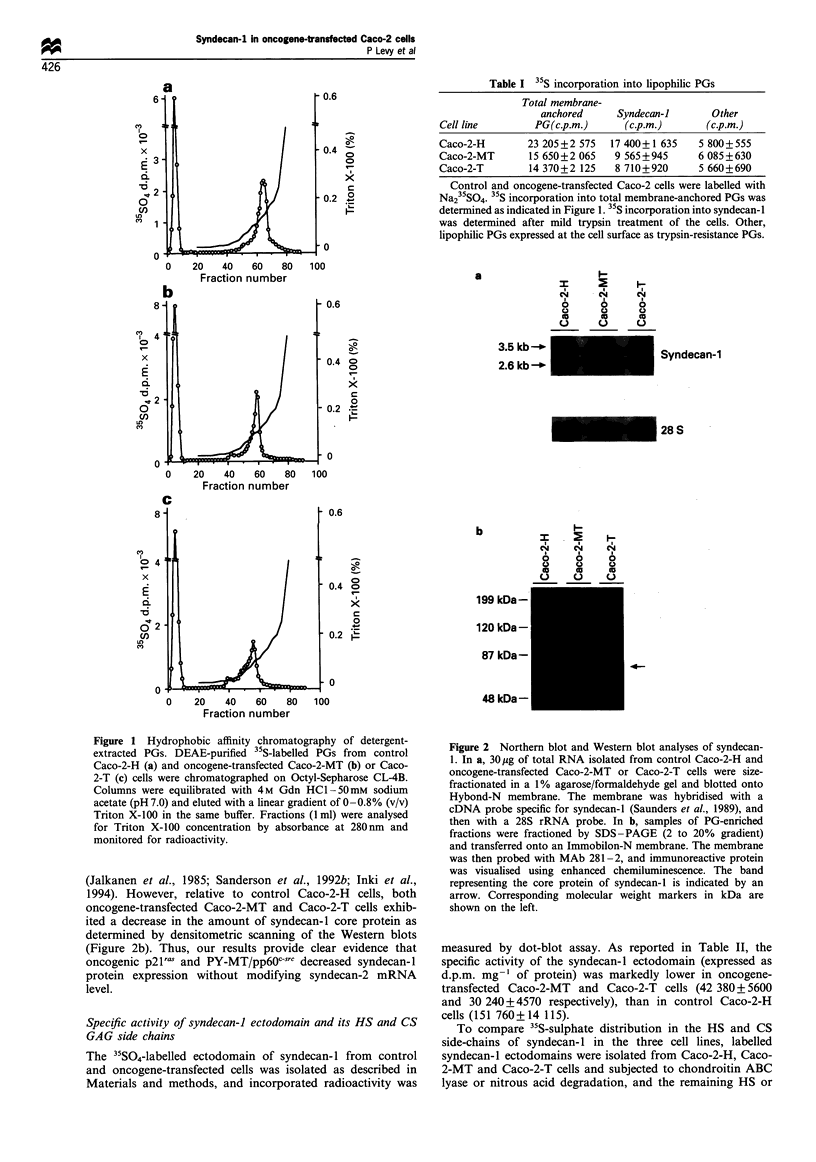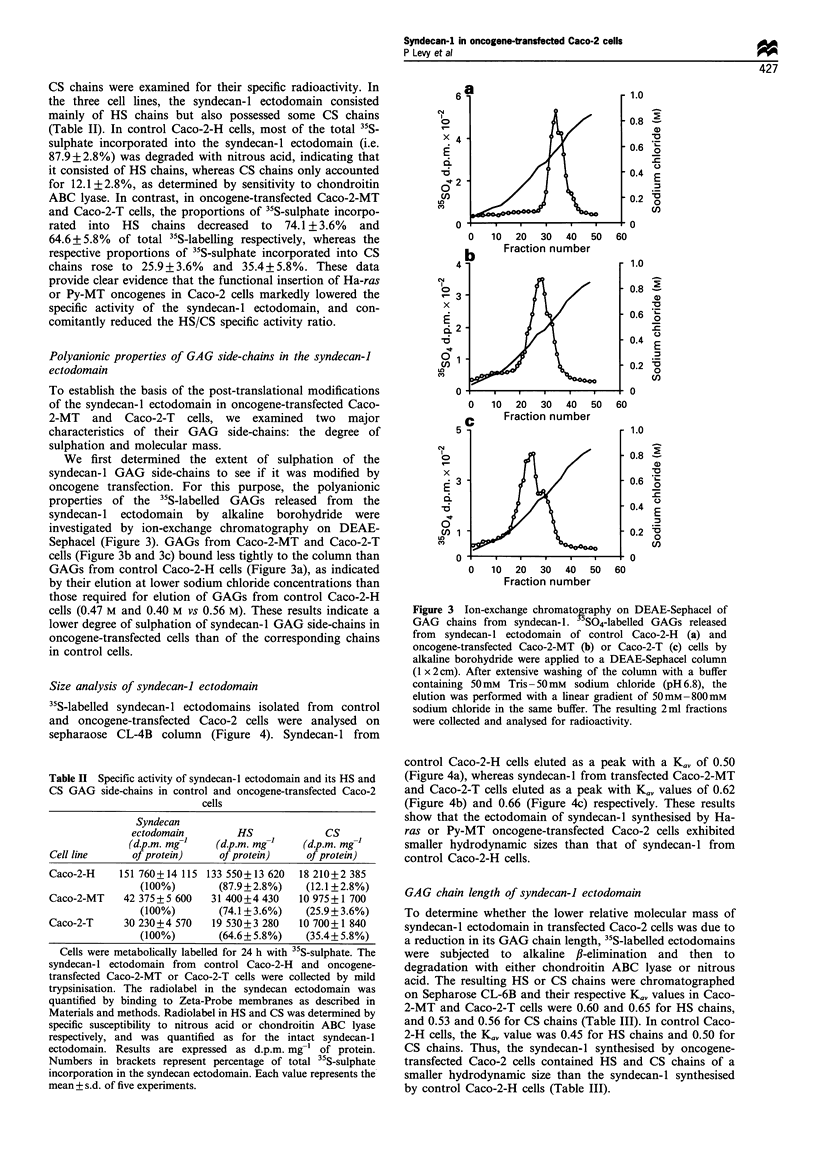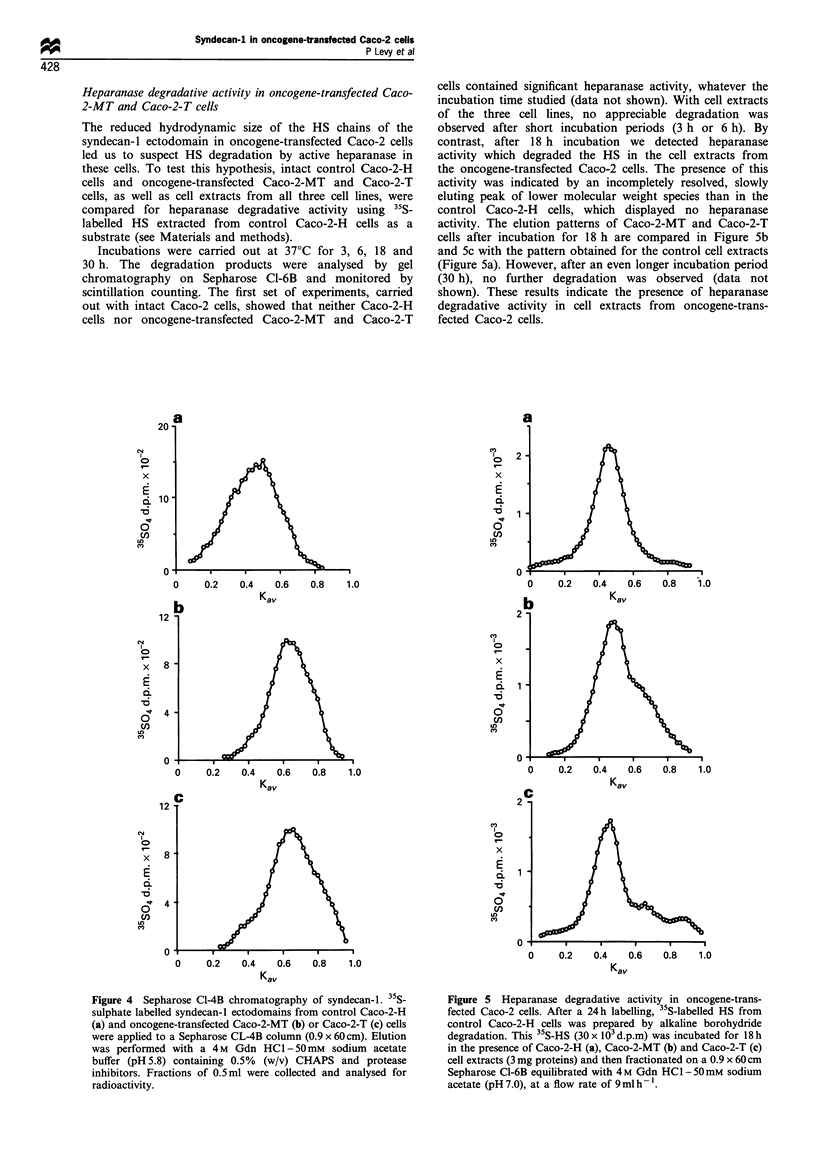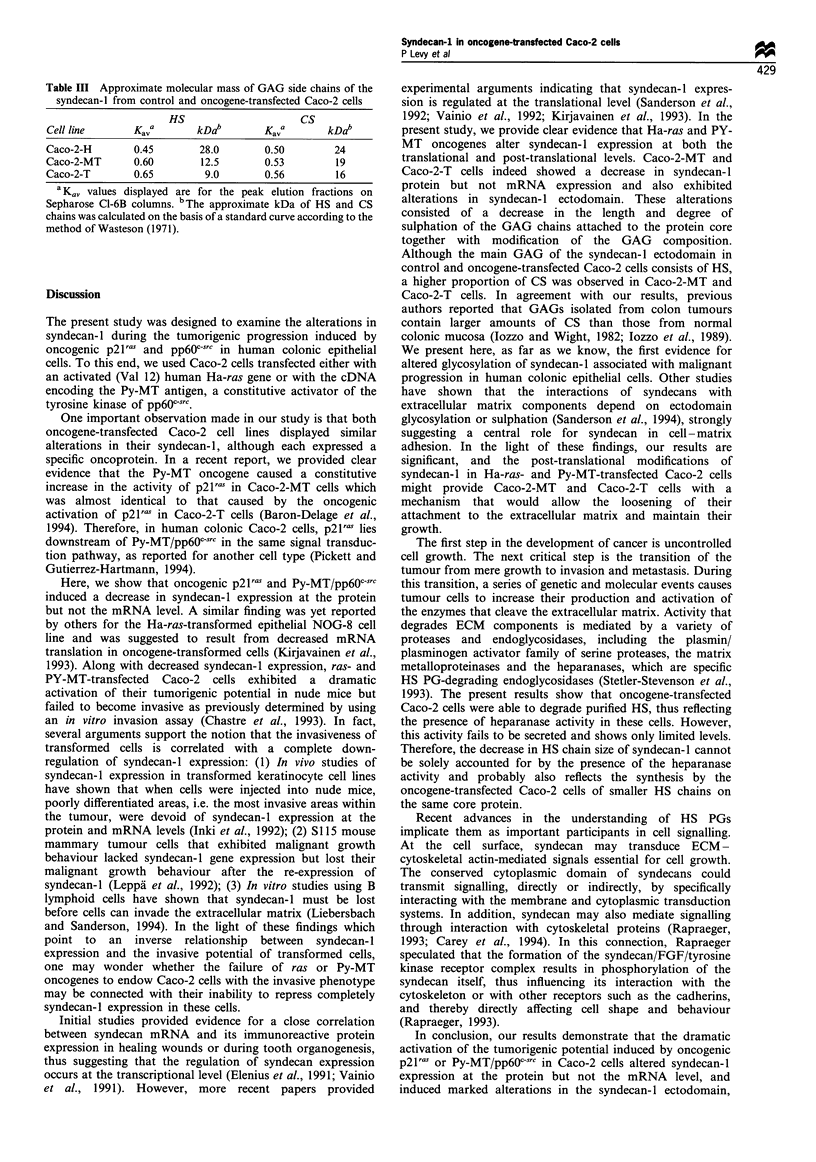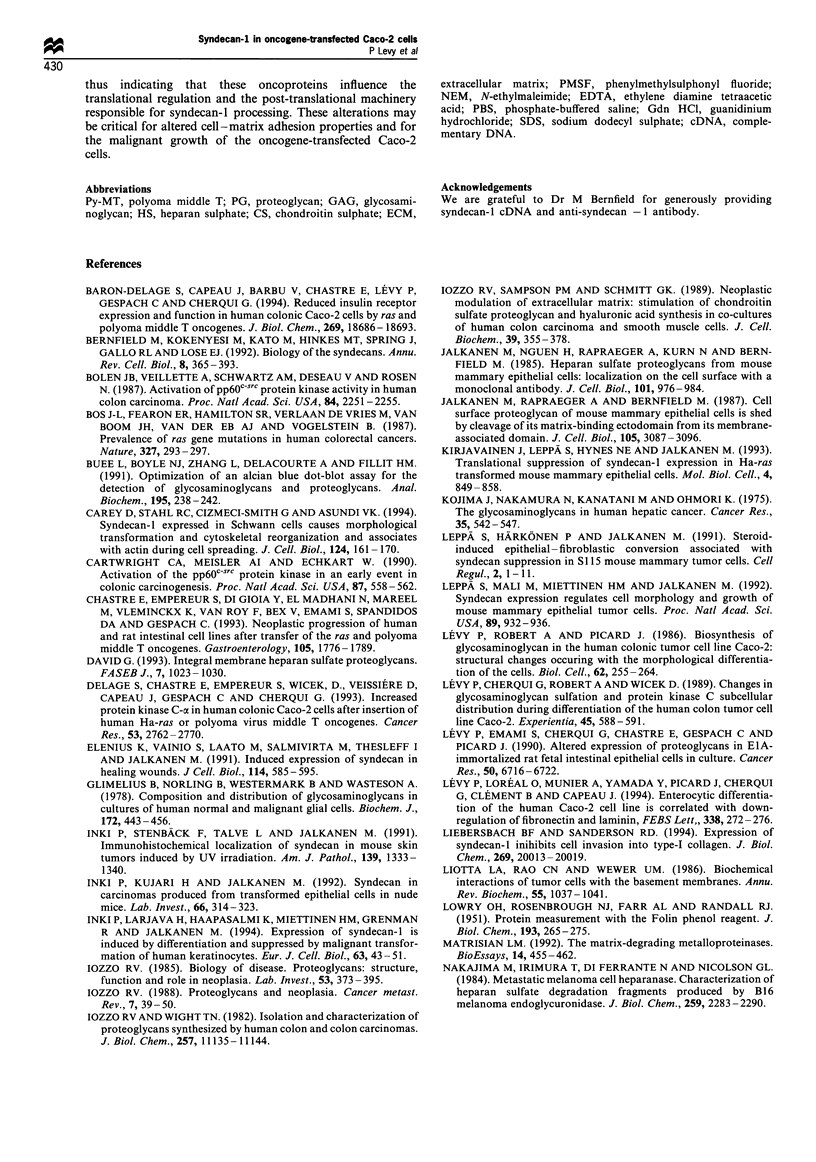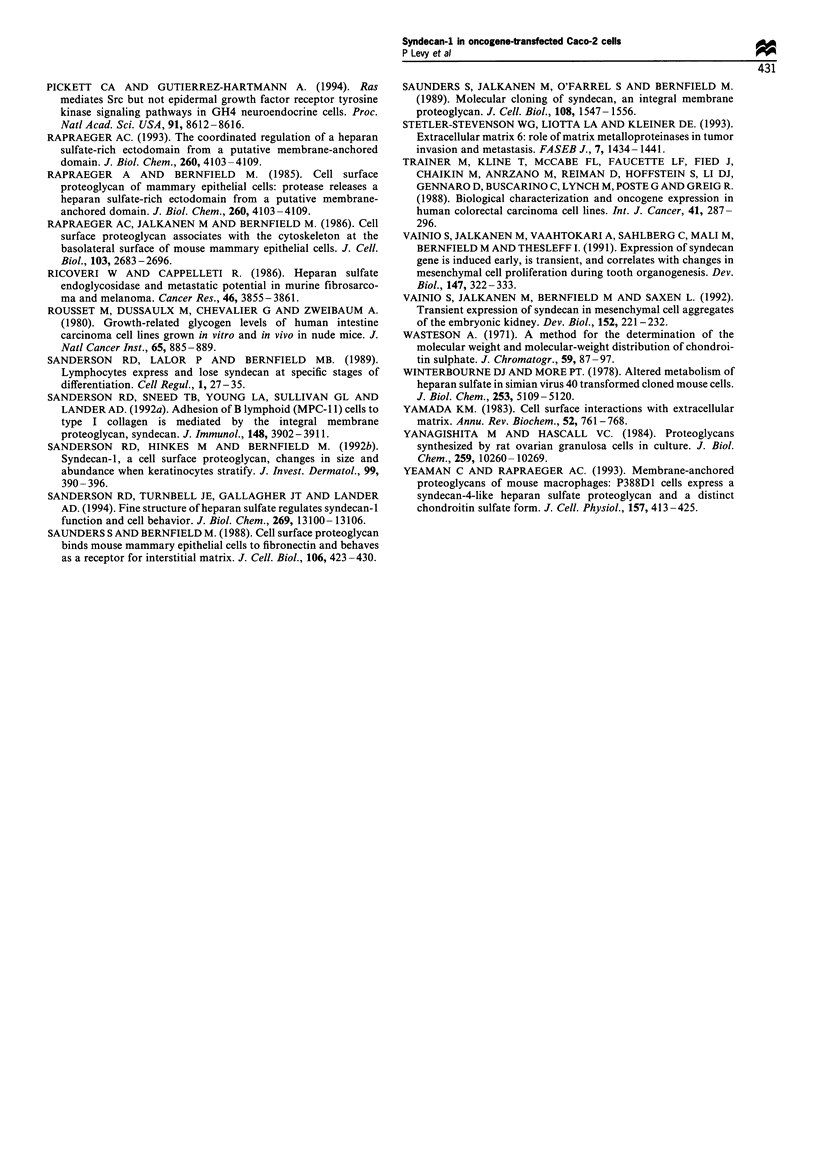# Syndecan-1 alterations during the tumorigenic progression of human colonic Caco-2 cells induced by human Ha-ras or polyoma middle T oncogenes.

**DOI:** 10.1038/bjc.1996.376

**Published:** 1996-08

**Authors:** P. Levy, A. Munier, S. Baron-Delage, Y. Di Gioia, C. Gespach, J. Capeau, G. Cherqui

**Affiliations:** Laboratoire de Biologie Cellulaire, INSERM-U.402, Faculté de Médecine Saint-Antoine, Paris, France.

## Abstract

**Images:**


British Journal of Cancer (1996) 74, 423-431
© 1996 Stockton Press All rights reserved 0007-0920/96 $12.00

### Syndecan-1 alterations during the tumorigenic progression of human colonic Caco-2 cells induced by human Ha-*ras* or polyoma middle T oncogenes

P Levy[1], A Munier[1], S Baron-Delage[1], Y Di Gioia[2], C Gespach[2], J Capeau[1] and G Cherqui[1]

*[1]Laboratoire de Biologie Cellulaire, INSERM-U.402, Faculté de Médecine Saint-Antoine, 27 rue Chaligny, 75571 Paris Cédex 12, France; [2]INSERM-U.55, Hôpital Saint-Antoine, 184 rue du Faubourg Saint-Antoine, 75571 Paris Cédex 12, France.*

**Summary** The products of *ras* and *src* proto-oncogenes are frequently activated in a constitutive state in human colorectal cancer. In this study we attempted to establish whether the tumorigenic progression induced by oncogenic activation of $p21^{ras}$ and $pp60^{c\text{-}src}$ in human colonic Caco-2 cells is associated with specific alterations of syndecan-1, a membrane-anchored proteoglycan playing a role in cell–matrix interaction and neoplastic growth control. To this end, we used Caco-2 cells made highly tumorigenic by transfection with an activated (Val 12) human Ha-*ras* gene or with the polyoma middle T (Py-MT) oncogene, a constitutive activator of $pp60^{c\text{-}src}$ tyrosine kinase activity. Compared with control vector-transfected Caco-2 cells, both oncogene-transfected cell lines (1) contained smaller amounts of membrane-anchored PGs; (2) exhibited decreased syndecan-1 expression at the protein but not the mRNA level; (3) synthesised $^{35}S$-labelled syndecan-1 with decreased specific activity; (4) produced a syndecan-1 ectodomain with a lower molecular mass and reduced GAG chain size and sulphation; and (5) expressed heparanase degradative activity. These results show that the dramatic activation of the tumorigenic potential induced by oncogenic $p21^{ras}$ or Py-MT/$pp60^{c\text{-}src}$ in Caco-2 cells is associated with marked alterations of syndecan-1 expression at the translational and post-translational levels.

**Keywords:** syndecan-1; Ha-*ras*; polyoma middle T; Caco-2 cells; tumorigenic progression

Human colorectal cancer, the second most common cause of death in developed countries, is now thought to result from a series of genetic alterations. The most frequent alterations consist of point mutations in members of the *ras* gene family (Bos *et al.*, 1987) which encode closely related $M_r$ 21 000 guanine nucleotide-binding proteins ($p21^{ras}$) or in activation of the pp60 phosphoprotein encoded by the proto-oncogene c-*src* (Bolen *et al.*, 1987; Cartwright *et al.*, 1990). The high frequency of these alterations strongly suggests that $p21^{ras}$ and $pp60^{c\text{-}src}$ oncoproteins, both of which are involved in growth factor signal transduction pathways, play a central role in human colon carcinogenesis.

Cell transformation is frequently associated with specific defects in the basement membrane, which play a critical role in tumour growth, invasion and metastasis (Liotta *et al.*, 1986; Yamada, 1983). These defects result mainly from the presence of specific enzymes involved in degradation of extracellular matrix constituents (Matrisian, 1992), and also from significant modifications in cell surface proteoglycans (PGs) (Iozzo, 1985). Specific qualitative and quantitative changes in PGs have been reported in various transformed cells. We previously demonstrated that the expression of PGs alters considerably in intestinal epithelial cells after oncogene immortalisation, representative of an early stage of neoplastic transformation (Lévy *et al.*, 1990). Elevated concentrations of hyaluronic acid have been demonstrated in breast carcinomas and human gliomas (Glimelius *et al.*, 1978), and increased amounts of chondroitin sulphate (CS) PGs have been shown in cancer cells from liver (Kojima *et al.*, 1975) and in colon cancer (Iozzo and Wight, 1982). Transformed cells are known to exhibit a reduced amount of undersulphated heparan sulphate (HS) (Winterbourne and Mora, 1978) and some authors have suggested that there is a relationship between decreased HS content and increased activity of heparanase, an enzyme which specifically degrades HS chains (Ricoveri and Capelleti, 1986; Nakajima *et al.*, 1984). Such alterations in the amount and degree of sulphation of HS may help to reduce cell matrix adhesion and thus favour tumour cell shedding from primary tumours (Iozzo, 1988).

Among the HS PGs, the syndecans, which constitute most of the transmembrane PGs, appear to have a profound influence on fundamental features of cell behaviour such as adhesion, matrix anchorage and growth control (David, 1993). The syndecan family consists of at least four members that differ considerably in their extracellular domains but exhibit high degrees of homology in their transmembrane and cytoplasmic domains (Bernfield *et al.*, 1992; Rapraeger, 1993). Syndecan-1, which is found mainly on the surfaces of epithelial cells in mature tissues, is the one that has been most thoroughly characterised so far (Saunders *et al.*, 1989). It plays a key role in the maintenance of the epithelial cell phenotype by anchoring cytoskeletal actin to the extracellular matrix (ECM) (Rapraeger *et al.*, 1986) and behaves like a matrix receptor, by binding to a variety of ECM components (Saunders and Bernfield, 1988; Sanderson *et al.*, 1992*a*). Despite the importance of the interaction between transformed cells and the surrounding matrix as a regulatory factor controlling the growth of these cells, there are only a few studies dealing with the effects of malignant transformation on syndecan-1. Loss of syndecan-1 expression occurs in experimental tumours induced by UV-irradiation of murine skin (Inki *et al.*, 1991), by glucocorticoid exposure of mouse mammary epithelial cells (Leppä *et al.*, 1991; Kirjavainen *et al.*, 1993) and in the malignant transformation of human keratinocytes (Inki *et al.*, 1994).

However, there is to our knowledge, no information about the changes of syndecan-1 associated with *ras* and *src* oncogene-mediated neoplastic colorectal transformation. To address this issue, we used human colon carcinoma cells Caco-2 which had been transfected with a plasmid vector recombined either with an activated (Val 12) human c-Ha-*ras* gene (Caco-2-T cells), or with the cDNA encoding the Py-MT antigen, a constitutive activator of the tyrosine kinase of $pp60^{c\text{-}src}$ (Caco-2 MT cells). Since parental Caco-2 cells are known to display a very low tumorigenicity (Rousset *et al.*, 1980; Trainer *et al.*, 1988), these *ras*- and Py-MT-transfected Caco-2 cells, recently shown to be highly tumorigenic in nude

Correspondence: P Levy
Received 4 May 1995; revised 3 February 1996; accepted 23 February 1996

mice (Chastre et al., 1993; Delage et al., 1993), provide very suitable models in which the two defects most frequently observed in human colon cancer are reproduced. In this study, we investigated whether the tumorigenic progression induced by oncogenic $p21^{ras}$ or Py-MT/$pp60^{c\text{-}src}$ in Caco-2 cells was associated with (1) a change in syndecan-1 expression; (2) alterations in syndecan-1 ectodomain glycosylation and sulphation; and (3) the expression of specific endoglycosidase degradative activity.

### Materials and methods

### Materials

Carrier-free $Na_2{}^{35}SO_4$ (270 mCi $mmol^{-1}$) was purchased from New England Nuclear, Boston, MA, USA. Chondroitin ABC lyase (EC 4.2.2.4) was obtained from Seikagaku Fine Chemicals, Tokyo, Japan. PD-10 columns, Sepharose CL-4B and Sepharose CL-6B were obtained from Pharmacia Fine Chemicals, Uppsala, Sweden. DEAE-Sephacel was from Whatman Biochemicals, Maidstone, Kent, UK. 3-[3-cholamido propyl)dimethyl-ammonio]-1-propane-sulphonate (CHAPS), phenylmethylsulphonyl fluoride (PMSF), *N*-ethylmaleimide (NEM) and benzamidine were from Calbiochem, San Diego, USA. Soybean trypsin inhibitor was from Sigma Chemical Company, St Louis, Missouri. Zeta-probe membranes were from Bio-Rad Laboratories, Richmond, USA and Immobilon-N membranes from Millipore, Bedford, MA, USA. All other reagents, obtained from Boehringer Mannheim, Indianapolis, IN, USA, were of the highest analytical grade.

### Cell lines

Human colon carcinoma cells Caco-2 were transfected by electroporation, as previously described (Chastre et al., 1993). Homer 6, a plasmid vector containing a MoMSVLTR-driven G418 resistance gene, which was recombined either with the cDNA encoding the Py-MT antigen (pHO6MT1) or with a mutated (Val 12) human Ha-*ras* gene (pHO6T1) was used for cell transfection. Caco-2 cells transfected with the plasmids Homer 6, pHO6MT1 or pHO6T1, were designated as Caco-2-H, Caco-2-MT and Caco-2-T cells respectively (Chastre et al., 1993; Delage et al., 1993). The oncogene-transfected Caco-2-MT and Caco-2-T cells are regularly verified for overexpression of PKC-α mRNA transcripts (Delage et al., 1993), which is taken as an index of maintenance of functional oncogenes in these cell lines.

Cells were routinely grown at 37°C on 100 mm diameter dishes in a humidified incubator equilibrated with 5% carbon dioxide using Dulbecco's modified Eagle medium (DMEM) (4.5 $gl^{-1}$ glucose) supplemented with 15% fetal calf serum, 2 mM glutamine, 100 units $ml^{-1}$ penicillin and 100 mg $ml^{-1}$ streptomycin. For all the assays reported below, the cells were harvested at confluency.

### Isolation of transmembrane PGs

Control Caco-2-H and oncogene-transfected Caco-2-MT and Caco-2-T cells were incubated with $Na_2{}^{35}SO_4$ (50 μCi $ml^{-1}$) for 24 h. After labelling, cell monolayers were washed twice in ice-cold calcium- and magnesium-free phosphate-buffered saline (PBS). An extraction protocol using a detergent cell lysis buffer was designed to obtain cellular fractions enriched in plasma membrane-associated PGs (Yanagishita and Hascall, 1984). Briefly, cells were extracted and sonicated in 8 M urea, 150 mM sodium chloride, 1% Triton X-100, 50 mM sodium acetate (pH 4.5), 5 mM EDTA, 5 mM benzamidine, 5 mM NEM and 1 mM PMSF (Yeaman and Rapraeger, 1993). The detergent extracts were then centrifuged at 600 × *g* to remove insoluble material. $^{35}S$-PGs in the supernatant were isolated by ion-exchange chromatography on DEAE-Sephacel. Columns were equilibrated with extraction buffer and eluted with a linear gradient of sodium chloride (0.05–1.0 M) in the same buffer. Fractions were assayed for $^{35}S$-radioactivity by scintillation counting in a LKB 1209 counter (ACS, Amersham, UK). The peak of radioactivity eluting in the salt gradient was dialysed extensively against 10 mM Tris-HCl (pH 7.4) containing 0.1% Triton X-100 and supplemented with PMSF at a final concentration of 0.1 mM (Yeaman and Rapraeger, 1993). Before hydrophobic chromatography, aliquots of the purified PGs were diluted 20-fold to reduce the detergent concentration.

### Hydrophobic affinity chromatography

The hydrophobic properties of the detergent-extracted $^{35}S$-PGs purified by ion-exchange chromatography were determined by gel chromatography on Octyl-Sepharose CL-4B. Columns (2 ml) were equilibrated with 4 M guanidinium hydrochloride (Gdn HCl)–50 mM sodium acetate (pH 7.0). After washing with the same buffer, columns were eluted with a 50 ml gradient of Triton X-100 (0–0.8%) in the Gdn HCl buffer. Fractions (1 ml) were analysed for the Triton X-100 concentration by absorbance at 280 nm and for $^{35}S$ radioactivity by liquid scintillation counting.

### Biochemical characterisation of syndecan-1

Expression of syndecan-1 in control and oncogene-transfected Caco-2 cells was analysed by Northern blot and immunoblot analyses. For Northern blot analysis, RNA samples containing 30 μg of total RNA were electrophoresed through 1% agarose–6% formaldehyde gels, blotted onto Hybond-N nylon membranes (Amersham) and hybridised with a mega-prime-labelled cDNA probe specific for syndecan-1 (Saunders et al., 1989). After hybridisation, blots were washed at 65°C to high stringency, (0.1 × SSC, 0.1% SDS), and exposed to Kodak X-Omat AR film with intensifying screens at −80°C. RNA was quantified and normalised by differential densitometric scanning of syndecan-1 bands *vs* the 28S rRNA bands (Lévy et al., 1994).

For immunoblot analysis, PG-enriched fractions were prepared from detergent extracts of cells and partially purified as indicated previously (Inki et al., 1994). Samples were fractionated by SDS–PAGE (2–20% gradient) in Tris borate buffer (Jalkanen et al., 1985), and electrophoretically transferred to Immobilon-N membranes. Membranes were then probed with MAb 281–2, an antibody which recognises the core protein of syndecan-1 (generous gift from Dr M Bernfield, Harvard Medical School, Boston, MA, USA). Detection of immunoreactive protein was performed with the RPN Amersham enhanced chemiluminescence Western blotting detection system.

### Isolation of the syndecan-1 ectodomain

The syndecan-1 ectodomain was isolated as described (Jalkanen et al., 1987). Briefly, after a 24 h incubation with $^{35}S$-sulphate, cells were scraped into cold PBS in the presence of proteinase inhibitors and centrifuged for 10 min at 300 *g*, and the pellet was exposed to trypsin (20 mg $ml^{-1}$) for 10 min on an ice bath. The reaction was stopped by adding 100 mg $ml^{-1}$ of soybean trypsin inhibitor. After a further centrifugation, the trypsin-cleavable ectodomain was obtained in the supernatant (Rapraeger and Bernfield, 1985).

### Quantification of syndecan-1

The syndecan-1 ectodomain was quantified by blotting on Zeta-Probe membranes. Briefly, aliquots of trypsin-released $^{35}S$-syndecan-1 ectodomain were brought to a final concentration of 8 M urea and equilibrated with loading buffer containing 10 mM Tris-HCl, 8 M urea, 0.1% Triton X-100 (pH 8.0) and were spotted onto Zeta-Probe membranes with a dot-blot microfiltration apparatus (Bio-Rad Laboratories), as described (Buee et al., 1991). After passive filtration, the membranes were extensively washed under vacuum with the loading buffer, then with 50 mM Tris HCl, 150 mM sodium chloride (pH 8.0) and finally with water. The dots were cut out and bound radiolabelled syndecan-1 ectodomain was quantified by scintillation counting.

Glycosaminoglycan (GAG) chains of syndecan-1 were characterised by their differential susceptibility to nitrous acid deminative cleavage or chondroitin ABC lyase degradation, as previously reported (Lévy *et al*., 1989). After nitrous acid degradation of the GAGs, CS chains remain, as judged by their sensitivity to chondroitinase digestion; alternatively, after chondroitin ABC lyase digestion of the GAGs, HS chains remain, identified by their susceptibility to nitrous acid degradation. After treatment, radiosulphate incorporation into HS and CS was quantified by spotting samples onto Zeta-Probe membranes as described above and by counting the corresponding membrane spots.

### *Gel filtration and ion-exchange chromatography*

Gel filtration of syndecan-1 ectodomain on Sepharose CL-4B (0.9 x 60 cm) was performed in 4 M Gdn HCl containing 50 mM sodium acetate, at a flow rate of 9 ml $h^{-1}$. Free GAG chains were produced from syndecan-1 ectodomain by $\beta$-elimination with alkaline borohydride, as previously described (Lévy *et al*., 1986). The HS and CS GAG chain sizes were determined after chromatography on Sepharose CL-6B (0.9 x 60 cm) by comparing the $K_{av}$ determined experimentally with a standard curve of log $M_r$ *vs* $K_{av}$ for GAG chains of various known molecular weights (Wasteson, 1971). Void and total volumes of the sizing columns were marked by blue dextran and phenol red respectively.

Ion-exchange chromatography of the GAG chains of the syndecan-1 ectodomain was performed on a DEAE-Sephacel column (1 x 3 cm). After washing the column with 50 mM Tris-HCl, 50 mM sodium chloride (pH 6.8), elution was performed with a linear 50 ml gradient of 50–800 mM sodium chloride in the same buffer under a constant flow rate of 5 ml $h^{-1}$. Fractions (2 ml) were collected. The gradient was determined by conductivity measurements of the fractions (radiometer) and radioactivity was measured.

### *Heparanase activity*

*Preparation of $^{35}$S-labelled HS from Caco-2-H cells* Caco-2-H cells were labelled with $^{35}$S-sulphate for 24 h. $^{35}$S-GAG were prepared by alkaline borohydride degradation as described above. GAGs were digested with chondroitin ABC lyase and the degradation products were removed by passing through a Sepharose Cl-6B column equilibrated with 4 M Gdn HCl containing 50 mM sodium acetate. The radioactive peak in the void volume was collected, dialysed and concentrated. This material treated with nitrous acid was completely degraded and was thus identified as pure HS.

*Cell extracts* Once confluent, cell monolayers of Caco-2-H, Caco-2-MT and Caco-2-T cells were washed 3 times with PBS and detached from the dish by gentle shaking at 37°C in the presence of calcium- and magnesium-free PBS containing 2 mM EDTA. Cells were collected by centrifugation at 900 x $g$ for 10 min at 4°C, washed 3 times with PBS and freeze-dried. The lyophilised cells were then dispersed in 0.1% Triton X-100 and sonicated for 30 s in an ice bath (Ricoveri and Cappelleti, 1986). Protein was assayed by the method of Lowry *et al*. (1951) using bovine serum albumin in 0.1% Triton X-100 as the standard. Approximately 30 x $10^3$ d.p.m. of $^{35}$S-HS isolated from Caco-2-H cells were added to cell extracts (3 mg proteins) from Caco-2-H, Caco-2-MT or Caco-2-T cells. All digestions were performed at 37°C in a 0.1 M sodium phosphate buffer (pH 6.0) containing 15 mM D-saccharic acid 1,4 lacton, an inhibitor of $\beta$-glucuronidase (Ricoveri and Cappelleti, 1986). Digestions were carried out in duplicate using a boiled extract as a control. At the end of incubation, the mixture was centrifuged at 10000 x $g$ for 5 min and the supernatant was applied to a Sepharose Cl-6B column, as described above.

*Intact cells* Equal amounts (30 x $10^3$ d.p.m.) of $^{35}$S-HS from control Caco-2-H cells were added to confluent cell monolayers of Caco-2-H, Caco-2-MT or Caco-2-T cells. The medium was buffered by adding 1 M Hepes (pH 7.4) to a final concentration of 10 mM. The cells were incubated for the indicated times with gentle-shaking. At the end of incubation, the medium was harvested, centrifuged at 10 000 x $g$ for 5 min and fractionated, as described for cell extracts.

### *Statistical analysis*

Results are means ± s.d. for the indicated numbers of experiments with different cell preparations.

### Results

### *Membrane-anchored PGs*

The PGs from control and oncogene-transfected Caco-2 cells were examined for the presence of lipophilic moieties by hydrophobic chromatography on Octyl-Sepharose CL-4B, a procedure currently used to identify PG species that are integral components of the plasma membrane. Chromatography of detergent-extracted PGs generated two sulphate-containing peaks (Figure 1). The first peak, which eluted before the beginning of the gradient, constituted the unbound fractions. The second peak, requiring a detergent-containing buffer to be displaced from the column, contained $^{35}$S-labelled PGs which bound to Octyl-Sepharose through hydrophobic interactions. This bound material comprised the lipophilic PGs. In control Caco-2-H cells (Figure 1a), 57.6% of the total $^{35}$S-labelled PGs bound to the column, and the hydrophobic PG fraction eluted at 0.14% Triton X-100. In contrast, in the oncogene-transfected Caco-2-MT cells (Figure 1b) and Caco-2-T cells (Figure 1c), only 40.5% and 37.2% of the total $^{35}$S-labelled PGs bound to the column, and the hydrophobic PG fractions eluted at Triton X-100 concentrations of 0.11% and 0.09% respectively. These results indicate that expression of Ha-*ras* or Py-MT oncogenes in Caco-2 cells induced a decrease in the amount of membrane-anchored PGs. As indicated in Table I, this decrease was selectively accounted for by a decrease in syndecan-1 since the amount of the other lipophilic PGs was not modified in oncogene-transfected Caco-2-MT and Caco-2T cells.

### *Syndecan-1 expression*

Among the membrane-anchored PGs, syndecan-1 is known to play a central role in the maintenance of the epithelial phenotype. We therefore examined the expression of this PG in Caco-2 cells to determine if it was altered by Ha-*ras* or Py-MT oncogenes. Figure 2 shows the results of Northern blot analysis using a cDNA probe specific for syndecan-1 (Saunders *et al*., 1989). In control Caco-2 cells, two mRNA transcripts of 2.6 and 3.4 kb were detected (Figure 2a), in accordance with the results reported for other cell types (Saunders *et al*., 1989; Kirjavainen *et al*., 1993; Inki *et al*., 1994). In oncogene-transfected Caco-2-MT and Caco-2-T cells, syndecan-1 mRNA transcripts were present in a relative abundance similar to that observed in Caco-2-H cells, as determined by densitometric scanning of the Northern blots and normalisation of the results with respect to the 28S signal (Figure 2a). These results indicate that neither Ha-*ras* nor PY-MT oncogenes modified the amount of syndecan-1 mRNA.

Figure 2b shows the results of immunoblot analysis using an anti-syndecan-1 antibody (Jalkanen *et al*., 1985). In all three cell lines, this antibody recognised a core protein of approximately 70 kDa, which is in accordance with the published molecular mass of syndecan-1 core protein

Figure 1 Hydrophobic affinity chromatography of detergent-extracted PGs. DEAE-purified $^{35}S$-labelled PGs from control Caco-2-H (a) and oncogene-transfected Caco-2-MT (b) or Caco-2-T (c) cells were chromatographed on Octyl-Sepharose CL-4B. Columns were equilibrated with 4 M Gdn HCl–50 mM sodium acetate (pH 7.0) and eluted with a linear gradient of 0–0.8% (v/v) Triton X-100 in the same buffer. Fractions (1 ml) were analysed for Triton X-100 concentration by absorbance at 280 nm and monitored for radioactivity.

(Jalkanen *et al.*, 1985; Sanderson *et al.*, 1992*b*; Inki *et al.*, 1994). However, relative to control Caco-2-H cells, both oncogene-transfected Caco-2-MT and Caco-2-T cells exhibited a decrease in the amount of syndecan-1 core protein as determined by densitometric scanning of the Western blots (Figure 2b). Thus, our results provide clear evidence that oncogenic $p21^{ras}$ and PY-MT/$pp60^{c\text{-}src}$ decreased syndecan-1 protein expression without modifying syndecan-2 mRNA level.

### *Specific activity of syndecan-1 ectodomain and its HS and CS GAG side chains*

The $^{35}SO_4$-labelled ectodomain of syndecan-1 from control and oncogene-transfected cells was isolated as described in Materials and methods, and incorporated radioactivity was

**Table I** $^{35}S$ incorporation into lipophilic PGs

| *Cell line* | *Total membrane-anchored PG(c.p.m.)* | *Syndecan-1 (c.p.m.)* | *Other (c.p.m.)* |
|---|---|---|---|
| Caco-2-H | 23 205±2 575 | 17 400±1 635 | 5 800±555 |
| Caco-2-MT | 15 650±2 065 | 9 565±945 | 6 085±630 |
| Caco-2-T | 14 370±2 125 | 8 710±920 | 5 660±690 |

Control and oncogene-transfected Caco-2 cells were labelled with $Na_2{}^{35}SO_4$. $^{35}S$ incorporation into total membrane-anchored PGs was determined as indicated in Figure 1. $^{35}S$ incorporation into syndecan-1 was determined after mild trypsin treatment of the cells. Other, lipophilic PGs expressed at the cell surface as trypsin-resistance PGs.

Figure 2 Northern blot and Western blot analyses of syndecan-1. In a, 30 μg of total RNA isolated from control Caco-2-H and oncogene-transfected Caco-2-MT or Caco-2-T cells were size-fractionated in a 1% agarose/formaldehyde gel and blotted onto Hybond-N membrane. The membrane was hybridised with a cDNA probe specific for syndecan-1 (Saunders *et al.*, 1989), and then with a 28S rRNA probe. In b, samples of PG-enriched fractions were fractioned by SDS–PAGE (2 to 20% gradient) and transferred onto an Immobilon-N membrane. The membrane was then probed with MAb 281–2, and immunoreactive protein was visualised using enhanced chemiluminescence. The band representing the core protein of syndecan-1 is indicated by an arrow. Corresponding molecular weight markers in kDa are shown on the left.

measured by dot-blot assay. As reported in Table II, the specific activity of the syndecan-1 ectodomain (expressed as d.p.m. $mg^{-1}$ of protein) was markedly lower in oncogene-transfected Caco-2-MT and Caco-2-T cells (42 380±5600 and 30 240±4570 respectively), than in control Caco-2-H cells (151 760±14 115).

To compare $^{35}S$-sulphate distribution in the HS and CS side-chains of syndecan-1 in the three cell lines, labelled syndecan-1 ectodomains were isolated from Caco-2-H, Caco-2-MT and Caco-2-T cells and subjected to chondroitin ABC lyase or nitrous acid degradation, and the remaining HS or

CS chains were examined for their specific radioactivity. In the three cell lines, the syndecan-1 ectodomain consisted mainly of HS chains but also possessed some CS chains (Table II). In control Caco-2-H cells, most of the total $^{35}$S-sulphate incorporated into the syndecan-1 ectodomain (i.e. 87.9±2.8%) was degraded with nitrous acid, indicating that it consisted of HS chains, whereas CS chains only accounted for 12.1±2.8%, as determined by sensitivity to chondroitin ABC lyase. In contrast, in oncogene-transfected Caco-2-MT and Caco-2-T cells, the proportions of $^{35}$S-sulphate incorporated into HS chains decreased to 74.1±3.6% and 64.6±5.8% of total $^{35}$S-labelling respectively, whereas the respective proportions of $^{35}$S-sulphate incorporated into CS chains rose to 25.9±3.6% and 35.4±5.8%. These data provide clear evidence that the functional insertion of Ha-*ras* or Py-MT oncogenes in Caco-2 cells markedly lowered the specific activity of the syndecan-1 ectodomain, and concomitantly reduced the HS/CS specific activity ratio.

### *Polyanionic properties of GAG side-chains in the syndecan-1 ectodomain*

To establish the basis of the post-translational modifications of the syndecan-1 ectodomain in oncogene-transfected Caco-2-MT and Caco-2-T cells, we examined two major characteristics of their GAG side-chains: the degree of sulphation and molecular mass.

We first determined the extent of sulphation of the syndecan-1 GAG side-chains to see if it was modified by oncogene transfection. For this purpose, the polyanionic properties of the $^{35}$S-labelled GAGs released from the syndecan-1 ectodomain by alkaline borohydride were investigated by ion-exchange chromatography on DEAE-Sephacel (Figure 3). GAGs from Caco-2-MT and Caco-2-T cells (Figure 3b and 3c) bound less tightly to the column than GAGs from control Caco-2-H cells (Figure 3a), as indicated by their elution at lower sodium chloride concentrations than those required for elution of GAGs from control Caco-2-H cells (0.47 M and 0.40 M *vs* 0.56 M). These results indicate a lower degree of sulphation of syndecan-1 GAG side-chains in oncogene-transfected cells than of the corresponding chains in control cells.

### *Size analysis of syndecan-1 ectodomain*

$^{35}$S-labelled syndecan-1 ectodomains isolated from control and oncogene-transfected Caco-2 cells were analysed on sepharaose CL-4B column (Figure 4). Syndecan-1 from

**Figure 3** Ion-exchange chromatography on DEAE-Sephacel of GAG chains from syndecan-1. $^{35}$SO$_4$-labelled GAGs released from syndecan-1 ectodomain of control Caco-2-H (**a**) and oncogene-transfected Caco-2-MT (**b**) or Caco-2-T (**c**) cells by alkaline borohydride were applied to a DEAE-Sephacel column (1×2 cm). After extensive washing of the column with a buffer containing 50 mM Tris–50 mM sodium chloride (pH 6.8), the elution was performed with a linear gradient of 50 mM–800 mM sodium chloride in the same buffer. The resulting 2 ml fractions were collected and analysed for radioactivity.

**Table II** Specific activity of syndecan-1 ectodomain and its HS and CS GAG side-chains in control and oncogene-transfected Caco-2 cells

| *Cell line* | *Syndecan ectodomain (d.p.m. mg$^{-1}$ of protein)* | *HS (d.p.m. mg$^{-1}$ of protein)* | *CS (d.p.m. mg$^{-1}$ of protein)* |
|---|---|---|---|
| Caco-2-H | 151 760±14 115 | 133 550±13 620 | 18 210±2 385 |
| | (100%) | (87.9±2.8%) | (12.1±2.8%) |
| Caco-2-MT | 42 375±5 600 | 31 400±4 430 | 10 975±1 700 |
| | (100%) | (74.1±3.6%) | (25.9±3.6%) |
| Caco-2-T | 30 230±4 570 | 19 530±3 280 | 10 700±1 840 |
| | (100%) | (64.6±5.8%) | (35.4±5.8%) |

Cells were metabolically labelled for 24 h with $^{35}$S-sulphate. The syndecan-1 ectodomain from control Caco-2-H and oncogene-transfected Caco-2-MT or Caco-2-T cells were collected by mild trypsinisation. The radiolabel in the syndecan ectodomain was quantified by binding to Zeta-Probe membranes as described in Materials and methods. Radiolabel in HS and CS was determined by specific susceptibility to nitrous acid or chondroitin ABC lyase respectively, and was quantified as for the intact syndecan-1 ectodomain. Results are expressed as d.p.m. mg$^{-1}$ of protein. Numbers in brackets represent percentage of total $^{35}$S-sulphate incorporation in the syndecan ectodomain. Each value represents the mean±s.d. of five experiments.

control Caco-2-H cells eluted as a peak with a $K_{av}$ of 0.50 (Figure 4a), whereas syndecan-1 from transfected Caco-2-MT and Caco-2-T cells eluted as a peak with $K_{av}$ values of 0.62 (Figure 4b) and 0.66 (Figure 4c) respectively. These results show that the ectodomain of syndecan-1 synthesised by Ha-*ras* or Py-MT oncogene-transfected Caco-2 cells exhibited smaller hydrodynamic sizes than that of syndecan-1 from control Caco-2-H cells.

### *GAG chain length of syndecan-1 ectodomain*

To determine whether the lower relative molecular mass of syndecan-1 ectodomain in transfected Caco-2 cells was due to a reduction in its GAG chain length, $^{35}$S-labelled ectodomains were subjected to alkaline $\beta$-elimination and then to degradation with either chondroitin ABC lyase or nitrous acid. The resulting HS or CS chains were chromatographed on Sepharose CL-6B and their respective $K_{av}$ values in Caco-2-MT and Caco-2-T cells were 0.60 and 0.65 for HS chains, and 0.53 and 0.56 for CS chains (Table III). In control Caco-2-H cells, the $K_{av}$ value was 0.45 for HS chains and 0.50 for CS chains. Thus, the syndecan-1 synthesised by oncogene-transfected Caco-2 cells contained HS and CS chains of a smaller hydrodynamic size than the syndecan-1 synthesised by control Caco-2-H cells (Table III).

*Heparanase degradative activity in oncogene-transfected Caco-2-MT and Caco-2-T cells*

The reduced hydrodynamic size of the HS chains of the syndecan-1 ectodomain in oncogene-transfected Caco-2 cells led us to suspect HS degradation by active heparanase in these cells. To test this hypothesis, intact control Caco-2-H cells and oncogene-transfected Caco-2-MT and Caco-2-T cells, as well as cell extracts from all three cell lines, were compared for heparanase degradative activity using $^{35}S$-labelled HS extracted from control Caco-2-H cells as a substrate (see Materials and methods).

Incubations were carried out at 37°C for 3, 6, 18 and 30 h. The degradation products were analysed by gel chromatography on Sepharose Cl-6B and monitored by scintillation counting. The first set of experiments, carried out with intact Caco-2 cells, showed that neither Caco-2-H cells nor oncogene-transfected Caco-2-MT and Caco-2-T cells contained significant heparanase activity, whatever the incubation time studied (data not shown). With cell extracts of the three cell lines, no appreciable degradation was observed after short incubation periods (3 h or 6 h). By contrast, after 18 h incubation we detected heparanase activity which degraded the HS in the cell extracts from the oncogene-transfected Caco-2 cells. The presence of this activity was indicated by an incompletely resolved, slowly eluting peak of lower molecular weight species than in the control Caco-2-H cells, which displayed no heparanase activity. The elution patterns of Caco-2-MT and Caco-2-T cells after incubation for 18 h are compared in Figure 5b and 5c with the pattern obtained for the control cell extracts (Figure 5a). However, after an even longer incubation period (30 h), no further degradation was observed (data not shown). These results indicate the presence of heparanase degradative activity in cell extracts from oncogene-transfected Caco-2 cells.

**Figure 4** Sepharose Cl-4B chromatography of syndecan-1. $^{35}S$-sulphate labelled syndecan-1 ectodomains from control Caco-2-H (**a**) and oncogene-transfected Caco-2-MT (**b**) or Caco-2-T (**c**) cells were applied to a Sepharose CL-4B column (0.9 × 60 cm). Elution was performed with a 4 M Gdn HCl – 50 mM sodium acetate buffer (pH 5.8) containing 0.5% (w/v) CHAPS and protease inhibitors. Fractions of 0.5 ml were collected and analysed for radioactivity.

**Figure 5** Heparanase degradative activity in oncogene-transfected Caco-2 cells. After a 24 h labelling, $^{35}S$-labelled HS from control Caco-2-H cells was prepared by alkaline borohydride degradation. This $^{35}S$-HS (30 × $10^3$ d.p.m) was incubated for 18 h in the presence of Caco-2-H (**a**), Caco-2-MT (**b**) and Caco-2-T (**c**) cell extracts (3 mg proteins) and then fractionated on a 0.9 × 60 cm Sepharose Cl-6B equilibrated with 4 M Gdn HCl – 50 mM sodium acetate (pH 7.0), at a flow rate of 9 ml $h^{-1}$.

**Table III** Approximate molecular mass of GAG side chains of the syndecan-1 from control and oncogene-transfected Caco-2 cells

| | HS | | CS | |
|---|---|---|---|---|
| *Cell line* | $K_{av}{}^{a}$ | *kDa*[b] | $K_{av}{}^{a}$ | *kDa*[b] |
| Caco-2-H | 0.45 | 28.0 | 0.50 | 24 |
| Caco-2-MT | 0.60 | 12.5 | 0.53 | 19 |
| Caco-2-T | 0.65 | 9.0 | 0.56 | 16 |

[a] $K_{av}$ values displayed are for the peak elution fractions on Sepharose Cl-6B columns. [b] The approximate kDa of HS and CS chains was calculated on the basis of a standard curve according to the method of Wasteson (1971).

### Discussion

The present study was designed to examine the alterations in syndecan-1 during the tumorigenic progression induced by oncogenic p21$^{ras}$ and pp60$^{c\text{-}src}$ in human colonic epithelial cells. To this end, we used Caco-2 cells transfected either with an activated (Val 12) human Ha-*ras* gene or with the cDNA encoding the Py-MT antigen, a constitutive activator of the tyrosine kinase of pp60$^{c\text{-}src}$.

One important observation made in our study is that both oncogene-transfected Caco-2 cell lines displayed similar alterations in their syndecan-1, although each expressed a specific oncoprotein. In a recent report, we provided clear evidence that the Py-MT oncogene caused a constitutive increase in the activity of p21$^{ras}$ in Caco-2-MT cells which was almost identical to that caused by the oncogenic activation of p21$^{ras}$ in Caco-2-T cells (Baron-Delage *et al.*, 1994). Therefore, in human colonic Caco-2 cells, p21$^{ras}$ lies downstream of Py-MT/pp60$^{c\text{-}src}$ in the same signal transduction pathway, as reported for another cell type (Pickett and Gutierrez-Hartmann, 1994).

Here, we show that oncogenic p21$^{ras}$ and Py-MT/pp60$^{c\text{-}src}$ induced a decrease in syndecan-1 expression at the protein but not the mRNA level. A similar finding was yet reported by others for the Ha-*ras*-transformed epithelial NOG-8 cell line and was suggested to result from decreased mRNA translation in oncogene-transformed cells (Kirjavainen *et al.*, 1993). Along with decreased syndecan-1 expression, *ras*- and PY-MT-transfected Caco-2 cells exhibited a dramatic activation of their tumorigenic potential in nude mice but failed to become invasive as previously determined by using an *in vitro* invasion assay (Chastre *et al.*, 1993). In fact, several arguments support the notion that the invasiveness of transformed cells is correlated with a complete down-regulation of syndecan-1 expression: (1) *In vivo* studies of syndecan-1 expression in transformed keratinocyte cell lines have shown that when cells were injected into nude mice, poorly differentiated areas, i.e. the most invasive areas within the tumour, were devoid of syndecan-1 expression at the protein and mRNA levels (Inki *et al.*, 1992); (2) S115 mouse mammary tumour cells that exhibited malignant growth behaviour lacked syndecan-1 gene expression but lost their malignant growth behaviour after the re-expression of syndecan-1 (Leppä *et al.*, 1992); (3) *In vitro* studies using B lymphoid cells have shown that syndecan-1 must be lost before cells can invade the extracellular matrix (Liebersbach and Sanderson, 1994). In the light of these findings which point to an inverse relationship between syndecan-1 expression and the invasive potential of transformed cells, one may wonder whether the failure of *ras* or Py-MT oncogenes to endow Caco-2 cells with the invasive phenotype may be connected with their inability to repress completely syndecan-1 expression in these cells.

Initial studies provided evidence for a close correlation between syndecan mRNA and its immunoreactive protein expression in healing wounds or during tooth organogenesis, thus suggesting that the regulation of syndecan expression occurs at the transcriptional level (Elenius *et al.*, 1991; Vainio *et al.*, 1991). However, more recent papers provided experimental arguments indicating that syndecan-1 expression is regulated at the translational level (Sanderson *et al.*, 1992; Vainio *et al.*, 1992; Kirjavainen *et al.*, 1993). In the present study, we provide clear evidence that Ha-*ras* and PY-MT oncogenes alter syndecan-1 expression at both the translational and post-translational levels. Caco-2-MT and Caco-2-T cells indeed showed a decrease in syndecan-1 protein but not mRNA expression and also exhibited alterations in syndecan-1 ectodomain. These alterations consisted of a decrease in the length and degree of sulphation of the GAG chains attached to the protein core together with modification of the GAG composition. Although the main GAG of the syndecan-1 ectodomain in control and oncogene-transfected Caco-2 cells consists of HS, a higher proportion of CS was observed in Caco-2-MT and Caco-2-T cells. In agreement with our results, previous authors reported that GAGs isolated from colon tumours contain larger amounts of CS than those from normal colonic mucosa (Iozzo and Wight, 1982; Iozzo *et al.*, 1989). We present here, as far as we know, the first evidence for altered glycosylation of syndecan-1 associated with malignant progression in human colonic epithelial cells. Other studies have shown that the interactions of syndecans with extracellular matrix components depend on ectodomain glycosylation or sulphation (Sanderson *et al.*, 1994), strongly suggesting a central role for syndecan in cell–matrix adhesion. In the light of these findings, our results are significant, and the post-translational modifications of syndecan-1 in Ha-*ras*- and Py-MT-transfected Caco-2 cells might provide Caco-2-MT and Caco-2-T cells with a mechanism that would allow the loosening of their attachment to the extracellular matrix and maintain their growth.

The first step in the development of cancer is uncontrolled cell growth. The next critical step is the transition of the tumour from mere growth to invasion and metastasis. During this transition, a series of genetic and molecular events causes tumour cells to increase their production and activation of the enzymes that cleave the extracellular matrix. Activity that degrades ECM components is mediated by a variety of proteases and endoglycosidases, including the plasmin/plasminogen activator family of serine proteases, the matrix metalloproteinases and the heparanases, which are specific HS PG-degrading endoglycosidases (Stetler-Stevenson *et al.*, 1993). The present results show that oncogene-transfected Caco-2 cells were able to degrade purified HS, thus reflecting the presence of heparanase activity in these cells. However, this activity fails to be secreted and shows only limited levels. Therefore, the decrease in HS chain size of syndecan-1 cannot be solely accounted for by the presence of the heparanase activity and probably also reflects the synthesis by the oncogene-transfected Caco-2 cells of smaller HS chains on the same core protein.

Recent advances in the understanding of HS PGs implicate them as important participants in cell signalling. At the cell surface, syndecan may transduce ECM–cytoskeletal actin-mediated signals essential for cell growth. The conserved cytoplasmic domain of syndecans could transmit signalling, directly or indirectly, by specifically interacting with the membrane and cytoplasmic transduction systems. In addition, syndecan may also mediate signalling through interaction with cytoskeletal proteins (Rapraeger, 1993; Carey *et al.*, 1994). In this connection, Rapraeger speculated that the formation of the syndecan/FGF/tyrosine kinase receptor complex results in phosphorylation of the syndecan itself, thus influencing its interaction with the cytoskeleton or with other receptors such as the cadherins, and thereby directly affecting cell shape and behaviour (Rapraeger, 1993).

In conclusion, our results demonstrate that the dramatic activation of the tumorigenic potential induced by oncogenic p21$^{ras}$ or Py-MT/pp60$^{c\text{-}src}$ in Caco-2 cells altered syndecan-1 expression at the protein but not the mRNA level, and induced marked alterations in the syndecan-1 ectodomain, thus indicating that these oncoproteins influence the translational regulation and the post-translational machinery responsible for syndecan-1 processing. These alterations may be critical for altered cell–matrix adhesion properties and for the malignant growth of the oncogene-transfected Caco-2 cells.

### Abbreviations

Py-MT, polyoma middle T; PG, proteoglycan; GAG, glycosaminoglycan; HS, heparan sulphate; CS, chondroitin sulphate; ECM, extracellular matrix; PMSF, phenylmethylsulphonyl fluoride; NEM, *N*-ethylmaleimide; EDTA, ethylene diamine tetraacetic acid; PBS, phosphate-buffered saline; Gdn HCl, guanidinium hydrochloride; SDS, sodium dodecyl sulphate; cDNA, complementary DNA.

### Acknowledgements

We are grateful to Dr M Bernfield for generously providing syndecan-1 cDNA and anti-syndecan −1 antibody.

### References

BARON-DELAGE S, CAPEAU J, BARBU V, CHASTRE E, LÉVY P, GESPACH C AND CHERQUI G. (1994). Reduced insulin receptor expression and function in human colonic Caco-2 cells by *ras* and polyoma middle T oncogenes. *J. Biol. Chem.*, **269**, 18686–18693.

BERNFIELD M, KOKENYESI M, KATO M, HINKES MT, SPRING J, GALLO RL AND LOSE EJ. (1992). Biology of the syndecans. *Annu. Rev. Cell. Biol.*, **8**, 365–393.

BOLEN JB, VEILLETTE A, SCHWARTZ AM, DESEAU V AND ROSEN N. (1987). Activation of pp60$^{c-src}$ protein kinase activity in human colon carcinoma. *Proc. Natl Acad. Sci. USA*, **84**, 2251–2255.

BOS J-L, FEARON ER, HAMILTON SR, VERLAAN DE VRIES M, VAN BOOM JH, VAN DER EB AJ AND VOGELSTEIN B. (1987). Prevalence of *ras* gene mutations in human colorectal cancers. *Nature*, **327**, 293–297.

BUEE L, BOYLE NJ, ZHANG L, DELACOURTE A AND FILLIT HM. (1991). Optimization of an alcian blue dot-blot assay for the detection of glycosaminoglycans and proteoglycans. *Anal. Biochem.*, **195**, 238–242.

CAREY D, STAHL RC, CIZMECI-SMITH G AND ASUNDI VK. (1994). Syndecan-1 expressed in Schwann cells causes morphological transformation and cytoskeletal reorganization and associates with actin during cell spreading. *J. Cell. Biol.*, **124**, 161–170.

CARTWRIGHT CA, MEISLER AI AND ECHKART W. (1990). Activation of the pp60$^{c-src}$ protein kinase in an early event in colonic carcinogenesis. *Proc. Natl Acad. Sci. USA*, **87**, 558–562.

CHASTRE E, EMPEREUR S, DI GIOIA Y, EL MADHANI N, MAREEL M, VLEMINCKX K, VAN ROY F, BEX V, EMAMI S, SPANDIDOS DA AND GESPACH C. (1993). Neoplastic progression of human and rat intestinal cell lines after transfer of the *ras* and polyoma middle T oncogenes. *Gastroenterology*, **105**, 1776–1789.

DAVID G. (1993). Integral membrane heparan sulfate proteoglycans. *FASEB J.*, **7**, 1023–1030.

DELAGE S, CHASTRE E, EMPEREUR S, WICEK, D., VEISSIÉRE D, CAPEAU J, GESPACH C AND CHERQUI G. (1993). Increased protein kinase C-α in human colonic Caco-2 cells after insertion of human Ha-*ras* or polyoma virus middle T oncogenes. *Cancer Res.*, **53**, 2762–2770.

ELENIUS K, VAINIO S, LAATO M, SALMIVIRTA M, THESLEFF I AND JALKANEN M. (1991). Induced expression of syndecan in healing wounds. *J Cell. Biol.*, **114**, 585–595.

GLIMELIUS B, NORLING B, WESTERMARK B AND WASTESON A. (1978). Composition and distribution of glycosaminoglycans in cultures of human normal and malignant glial cells. *Biochem. J.*, **172**, 443–456.

INKI P, STENBÄCK F, TALVE L AND JALKANEN M. (1991). Immunohistochemical localization of syndecan in mouse skin tumors induced by UV irradiation. *Am. J. Pathol.*, **139**, 1333–1340.

INKI P, KUJARI H AND JALKANEN M. (1992). Syndecan in carcinomas produced from transformed epithelial cells in nude mice. *Lab. Invest.*, **66**, 314–323.

INKI P, LARJAVA H, HAAPASALMI K, MIETTINEN HM, GRENMAN R AND JALKANEN M. (1994). Expression of syndecan-1 is induced by differentiation and suppressed by malignant transformation of human keratinocytes. *Eur. J. Cell. Biol.*, **63**, 43–51.

IOZZO RV. (1985). Biology of disease. Proteoglycans: structure, function and role in neoplasia. *Lab. Invest.*, **53**, 373–395.

IOZZO RV. (1988). Proteoglycans and neoplasia. *Cancer metast. Rev.*, **7**, 39–50.

IOZZO RV AND WIGHT TN. (1982). Isolation and characterization of proteoglycans synthesized by human colon and colon carcinomas. *J. Biol. Chem.*, **257**, 11135–11144.

IOZZO RV, SAMPSON PM AND SCHMITT GK. (1989). Neoplastic modulation of extracellular matrix: stimulation of chondroitin sulfate proteoglycan and hyaluronic acid synthesis in co-cultures of human colon carcinoma and smooth muscle cells. *J. Cell. Biochem.*, **39**, 355–378.

JALKANEN M, NGUEN H, RAPRAEGER A, KURN N AND BERNFIELD M. (1985). Heparan sulfate proteoglycans from mouse mammary epithelial cells: localization on the cell surface with a monoclonal antibody. *J. Cell. Biol.*, **101**, 976–984.

JALKANEN M, RAPRAEGER A AND BERNFIELD M. (1987). Cell surface proteoglycan of mouse mammary epithelial cells is shed by cleavage of its matrix-binding ectodomain from its membrane-associated domain. *J. Cell. Biol.*, **105**, 3087–3096.

KIRJAVAINEN J, LEPPÄ S, HYNES NE AND JALKANEN M. (1993). Translational suppression of syndecan-1 expression in Ha-*ras* transformed mouse mammary epithelial cells. *Mol. Biol. Cell.*, **4**, 849–858.

KOJIMA J, NAKAMURA N, KANATANI M AND OHMORI K. (1975). The glycosaminoglycans in human hepatic cancer. *Cancer Res.*, **35**, 542–547.

LEPPÄ S, HÄRKÖNEN P AND JALKANEN M. (1991). Steroid-induced epithelial–fibroblastic conversion associated with syndecan suppression in S115 mouse mammary tumor cells. *Cell Regul.*, **2**, 1–11.

LEPPÄ S, MALI M, MIETTINEN HM AND JALKANEN M. (1992). Syndecan expression regulates cell morphology and growth of mouse mammary epithelial tumor cells. *Proc. Natl Acad. Sci. USA*, **89**, 932–936.

LÉVY P, ROBERT A AND PICARD J. (1986). Biosynthesis of glycosaminoglycan in the human colonic tumor cell line Caco-2: structural changes occuring with the morphological differentiation of the cells. *Biol. Cell.*, **62**, 255–264.

LÉVY P, CHERQUI G, ROBERT A AND WICEK D. (1989). Changes in glycosaminoglycan sulfation and protein kinase C subcellular distribution during differentiation of the human colon tumor cell line Caco-2. *Experientia*, **45**, 588–591.

LÉVY P, EMAMI S, CHERQUI G, CHASTRE E, GESPACH C AND PICARD J. (1990). Altered expression of proteoglycans in E1A-immortalized rat fetal intestinal epithelial cells in culture. *Cancer Res.*, **50**, 6716–6722.

LÉVY P, LORÉAL O, MUNIER A, YAMADA Y, PICARD J, CHERQUI G, CLÉMENT B AND CAPEAU J. (1994). Enterocytic differentiation of the human Caco-2 cell line is correlated with down-regulation of fibronectin and laminin, *FEBS Lett*,, **338**, 272–276.

LIEBERSBACH BF AND SANDERSON RD. (1994). Expression of syndecan-1 inihibits cell invasion into type-I collagen. *J. Biol. Chem.*, **269**, 20013–20019.

LIOTTA LA, RAO CN AND WEWER UM. (1986). Biochemical interactions of tumor cells with the basement membranes. *Annu. Rev. Biochem.*, **55**, 1037–1041.

LOWRY OH, ROSENBROUGH NJ, FARR AL AND RANDALL RJ. (1951). Protein measurement with the Folin phenol reagent. *J. Biol. Chem.*, **193**, 265–275.

MATRISIAN LM. (1992). The matrix-degrading metalloproteinases. *BioEssays*, **14**, 455–462.

NAKAJIMA M, IRIMURA T, DI FERRANTE N AND NICOLSON GL. (1984). Metastatic melanoma cell heparanase. Characterization of heparan sulfate degradation fragments produced by B16 melanoma endoglycuronidase. *J. Biol. Chem.*, **259**, 2283–2290.

PICKETT CA AND GUTIERREZ-HARTMANN A. (1994). *Ras* mediates Src but not epidermal growth factor receptor tyrosine kinase signaling pathways in GH4 neuroendocrine cells. *Proc. Natl Acad. Sci. USA*, **91,** 8612–8616.

RAPRAEGER AC. (1993). The coordinated regulation of a heparan sulfate-rich ectodomain from a putative membrane-anchored domain. *J. Biol. Chem.*, **260,** 4103–4109.

RAPRAEGER A AND BERNFIELD M. (1985). Cell surface proteoglycan of mammary epithelial cells: protease releases a heparan sulfate-rich ectodomain from a putative membrane-anchored domain. *J. Biol. Chem.*, **260,** 4103–4109.

RAPRAEGER AC, JALKANEN M AND BERNFIELD M. (1986). Cell surface proteoglycan associates with the cytoskeleton at the basolateral surface of mouse mammary epithelial cells. *J. Cell. Biol.*, **103,** 2683–2696.

RICOVERI W AND CAPPELLETI R. (1986). Heparan sulfate endoglycosidase and metastatic potential in murine fibrosarcoma and melanoma. *Cancer Res.*, **46,** 3855–3861.

ROUSSET M, DUSSAULX M, CHEVALIER G AND ZWEIBAUM A. (1980). Growth-related glycogen levels of human intestine carcinoma cell lines grown *in vitro* and *in vivo* in nude mice. *J. Natl Cancer Inst.*, **65,** 885–889.

SANDERSON RD, LALOR P AND BERNFIELD MB. (1989). Lymphocytes express and lose syndecan at specific stages of differentiation. *Cell Regul.*, **1,** 27–35.

SANDERSON RD, SNEED TB, YOUNG LA, SULLIVAN GL AND LANDER AD. (1992*a*). Adhesion of B lymphoid (MPC-11) cells to type I collagen is mediated by the integral membrane proteoglycan, syndecan. *J. Immunol.*, **148,** 3902–3911.

SANDERSON RD, HINKES M AND BERNFIELD M. (1992*b*). Syndecan-1, a cell surface proteoglycan, changes in size and abundance when keratinocytes stratify. *J. Invest. Dermatol.*, **99,** 390–396.

SANDERSON RD, TURNBELL JE, GALLAGHER JT AND LANDER AD. (1994). Fine structure of heparan sulfate regulates syndecan-1 function and cell behavior. *J. Biol. Chem.*, **269,** 13100–13106.

SAUNDERS S AND BERNFIELD M. (1988). Cell surface proteoglycan binds mouse mammary epithelial cells to fibronectin and behaves as a receptor for interstitial matrix. *J. Cell. Biol.*, **106,** 423–430.

SAUNDERS S, JALKANEN M, O'FARREL S AND BERNFIELD M. (1989). Molecular cloning of syndecan, an integral membrane proteoglycan. *J. Cell. Biol.*, **108,** 1547–1556.

STETLER-STEVENSON WG, LIOTTA LA AND KLEINER DE. (1993). Extracellular matrix 6: role of matrix metalloproteinases in tumor invasion and metastasis. *FASEB J.*, **7,** 1434–1441.

TRAINER M, KLINE T, MCCABE FL, FAUCETTE LF, FIED J, CHAIKIN M, ANRZANO M, REIMAN D, HOFFSTEIN S, LI DJ, GENNARO D, BUSCARINO C, LYNCH M, POSTE G AND GREIG R. (1988). Biological characterization and oncogene expression in human colorectal carcinoma cell lines. *Int. J. Cancer*, **41,** 287–296.

VAINIO S, JALKANEN M, VAAHTOKARI A, SAHLBERG C, MALI M, BERNFIELD M AND THESLEFF I. (1991). Expression of syndecan gene is induced early, is transient, and correlates with changes in mesenchymal cell proliferation during tooth organogenesis. *Dev. Biol.*, **147,** 322–333.

VAINIO S, JALKANEN M, BERNFIELD M AND SAXEN L. (1992). Transient expression of syndecan in mesenchymal cell aggregates of the embryonic kidney. *Dev. Biol.*, **152,** 221–232.

WASTESON A. (1971). A method for the determination of the molecular weight and molecular-weight distribution of chondroitin sulphate. *J. Chromatogr.*, **59,** 87–97.

WINTERBOURNE DJ AND MORE PT. (1978). Altered metabolism of heparan sulfate in simian virus 40 transformed cloned mouse cells. *J. Biol. Chem.*, **253,** 5109–5120.

YAMADA KM. (1983). Cell surface interactions with extracellular matrix. *Annu. Rev. Biochem.*, **52,** 761–768.

YANAGISHITA M AND HASCALL VC. (1984). Proteoglycans synthesized by rat ovarian granulosa cells in culture. *J. Biol. Chem.*, **259,** 10260–10269.

YEAMAN C AND RAPRAEGER AC. (1993). Membrane-anchored proteoglycans of mouse macrophages: P388D1 cells express a syndecan-4-like heparan sulfate proteoglycan and a distinct chondroitin sulfate form. *J. Cell. Physiol.*, **157,** 413–425.